# The prevalence, temporal trends, and geographical distribution of HIV-1 subtypes among men who have sex with men in China: A systematic review and meta-analysis

**DOI:** 10.1017/S0950268818003400

**Published:** 2019-02-19

**Authors:** Yueqi Yin, Yuxiang Liu, Jing Zhu, Xiang Hong, Rui Yuan, Gengfeng Fu, Ying Zhou, Bei Wang

**Affiliations:** 1 Department of Epidemiology and Health Statistics, Key Laboratory of Environmental Medicine Engineering of Ministry of Education, School of Public Health, Southeast University, Nanjing, China; 2 Huzhou Center for Disease Control and Prevention, Huzhou, Zhejiang, China; 3 Department of HIV/STD Prevention and Control, Jiangsu Provincial Center for Disease Prevention and Control, Nanjing 210009, China

**Keywords:** China, HIV-1, men who have sex with men, subtypes

## Abstract

The aim of this meta-analysis was to provide a comprehensive overview of human immunodeficiency virus (HIV)-1 subtypes and to investigate temporal and geographical trends of the HIV-1 epidemic among men who have sex with men (MSM) in China. Chinese and English articles published between January 2007 and December 2017 were systematically searched. Pooled HIV-1 prevalence was calculated, and its stability was analysed using sensitivity analysis. Subgroups were based on study time period, sampling area and prevalence. Publication bias was measured using Funnel plot and Egger's test. A total of 68 independent studies that included HIV-1 molecular investigations were eligible for meta-analysis. Circulating recombinant form (CRF) 01_AE (57.36%, 95% confidence interval (CI) 53.76–60.92) was confirmed as the most prevalent HIV-1 subtype among MSM in China. Subgroup analysis for time period found that CRF01_AE steadily increased prior to 2012 but decreased during 2012–2016. Further whereas CRF07_BC increased over time, B/B′ decreased over time. CRF55_01B has increased in recent years, with higher pooled estimated rate in Guangdong (12.22%, 95% CI 10.34–13.17) and Fujian (8.65%, 95% CI 4.98–13.17) provinces. The distribution of HIV-1 subtypes among MSM in China has changed across different regions and periods. HIV-1 strains in MSM are becoming more complex. Long-term molecular monitoring in this population remains necessary for HIV-1 epidemic control and prevention.

## Introduction

Human immunodeficiency virus/acquired immunodeficiency syndrome (HIV/AIDS) is still a serious global health problem. According to the Joint United Nations Programme on HIV/AIDS (UNAIDS), approximately 36.7 million (30.8–42.9) people globally were living with HIV-1 in 2016 [1]. The Chinese Center for Disease Control and Prevention (CDC) reported that there were 758 610 people infected with HIV by the end of 2017, including 134 512 people newly infected with HIV in that year [2].

Since the first confirmed HIV infection in 1985 [3], the spread of the disease has continued over 30 years in China, and enormous changes in the transmission mode have also occurred. Similar to other Asian countries, the epidemic of HIV-1 in China mainly involved intravenous drug users (IDUs) in the 1980's [4] and commercial blood donors in the 1990's [5]. The implementation of *Regulations on the administration of blood products* and *Anti-Drug Law of the People's Republic of China* have had a remarkable effect, and the HIV transmission mode has shifted to sexual contact, especially in the men who have sex with men (MSM) population [6]. MSM in Asia are 19 times more likely to be living with HIV than the general population [7]. According to data from the Chinese Ministry of Health and UNAIDS, MSM comprised 2.5% of all cases in 2006 and reached 25.8% in 2014 [8] and 25.5% (*N* = 34 358) of new HIV infections were among MSM in 2017 [2]. In some developed areas and large cities, MSM account for more than 50% of all HIV cases: 54.3% in Zhejiang [9], 74.0% in Hebei [10], 76.6% in Tianjin [11]; these rates are even higher among young people living with HIV/AIDS. MSM have become critical to China's HIV/AIDS prevention efforts.

In China, MSM generally have more than one sexual partner [12] and have lower rates of condom use [13]. Moreover, HIV is a highly variable virus [14]. Due to this high-risk behaviour and viral characteristics, the genetics of HIV-1 have become increasingly complex among MSM [15, 16]. The initial dominant subtype of HIV-1 in MSM was B/B′, but this has been substituted by the circulating recombinant form (CRF) 01_AE [17]. Subsequently, subtypes of HIV-1 among Chinese MSM have become very complicated, and more and more CRFs and unique recombinant forms (URFs) have been detected in this population. Subtype CRF55_01B was identified at the end of 2012 in China [18], and subtype CRF59_01B was identified at the beginning of 2013 among MSM in North-eastern China [19]. In addition, a novel drug-resistant HIV-1 recombinant CRF01_AE/B/C was identified in 2015 among MSM in Beijing [20], and CRF01_AE/CRF07_BC was detected in 2014 among MSM in Jilin province [21].

The epidemic of HIV in MSM is a concern among many researchers, with changes occurring continuously and in many different location. Comprehensive research examining MSM in China has shown that the pooled rate of subtype B/B′ and CRF01_AE in 2013 was 28.25% and 53.46% [22], which then declined in 2015 to 17.74% and 51.28% [23], respectively. Another study showed that from 2009 to 2014, the pooled rate of subtype B/B′ and CRF01_AE was 9.8% and 45.3%, respectively [24], which is different from the two previously cited studies. In some developed areas, the prevalence of different HIV-1 subtypes has also demonstrated change according to sampling time. From 2006 to 2012 in Shenzhen, the prevalence of HIV-1 subtype B decreased from 37.5% to 5.7% and that of CRF01_AE decreased from 50.0% to 32.3%; however, the prevalence of CRF07_BC increased from 12.5% to 43.2% and that of CRF55_01B increased from 0% to 16.0% [25]. Between 2012 and 2015 in Jiangsu province, the prevalence of HIV-1 subtype CRF01_AE decreased from 56.5% to 49.3%, and that of CRF07_BC increased from 13.0% to 39.4% [26]. In the span of a few years, the prevalence of multiple HIV-1 subtypes have changed considerably. The characteristics of the current epidemic among MSM in China are unclear. The purpose of this systematic review and meta-analysis was to evaluate the available literature in China and summarise the prevalence of HIV-1 subtypes among MSM, to explore the temporal trends and geographical patterns of HIV-1 subtypes and to better understand the HIV-1 epidemic among MSM in China.

## Methods

### Search strategy

In this systematic review and meta-analysis, we searched the following English and Chinese literature databases: PubMed, Web of Science, China National Knowledge Infrastructure (CNKI), Wanfang Data, Chinese Scientific Journals Fulltext Database (CQVIP) and China Biological Medicine (CBM). These literatures published up to 31 December 2017 with restriction on language in English and Chinese. Keywords for searching potential articles included (‘HIV’ or ‘AIDS’ or ‘human immunodeficiency virus’ or ‘acquired immunodeficiency syndrome’) and (‘China’ or ‘Chinese’) and (‘MSM’ or ‘the man who have sex with man’ or ‘gay’ or ‘same sex’) and (‘subtype’ or ‘genotype’ or ‘molecular epidemiology’), adjusted for different database. Systematic reviews with a similar theme were searched to determine if additional studies or unpublished data were reported. The reference of all included studies and systematic reviews were screened to identify additional eligible studies.

### Selection criteria

Published studies were enrolled if they met the following criteria: (1) the theme of studies were HIV-1 molecular epidemiology or included reports of HIV-1 subtypes; (2) the studies included MSM in China who were positive for HIV-1; (3) for studies that did not include MSM only, the transmission routes was classified clearly and included MSM; (4) subtyping method was clear and specific, using standard laboratory methods mainly consisting of specific genome region usually *env*, *gag* and/or *pol* amplification with polymerase chain reaction (PCR) and determine subtype using PCR products sequencing and phylogenetic tree analysis; (5) the prevalence of different HIV-1 subtypes among MSM must be available and (6) sample size of the study was more than 10.

The following types of reports were excluded: (1) review papers, meta-analysis, case reports, letters, conference abstracts, presentation and studies of single specific subtype; (2) duplication publication: same data source and same study period (worse quality excluded), overlapping sample collection time (earlier time excluded) and same data published in both Chinese and English (Chinese excluded) and (3) studies reporting conflicting data.

### Data extraction

Two reviewers independently extracted information from full texts of potentially eligible articles, including first author, publication year, study period, study location, case source, sample size, laboratory testing method for HIV-1 genotyping, the number of successfully identified HIV-1 subtypes and the frequency of different HIV-1 subtypes. All disagreements were reconciled by discussion between two authors until settled. Studies were classified into four locations according to their connection of geographical position: East, Northeast, North and Southwest. Based on the availability of data, studies were further classified into four 3-year time periods of data collection: ⩽2007, 2008–2010, 2011–2013 and ⩾2014.

### Data analysis

Estimates of the pooled proportion of different HIV-1 subtypes were computed using Freeman–Tukey double arcsine transformation to stabilise the variances [22, 23]. Heterogeneity was measured using Cochran *Q*-test *P* value and *I*^2^ statistics. *P* < 0.1 represented heterogeneity is statistically significant, *I*^2^ ranges from 0% to 100% suggested the levels of heterogeneity from low to high [27, 28]. Both of the fixed-effects and random-effects models were used to estimate the pooled prevalence and their corresponding 95% confidence intervals (CI). When *I*^2^ statistics was more than 50%, the random-effects model will be used. Funnel plot and Egger test were used to test the potential publication bias. All analyses were carried out by meta package of R version 3.4.1 (2017, The R Foundation for Statistical Computing Platform).

## Results

### Study identification and description

**Fig. 1.**
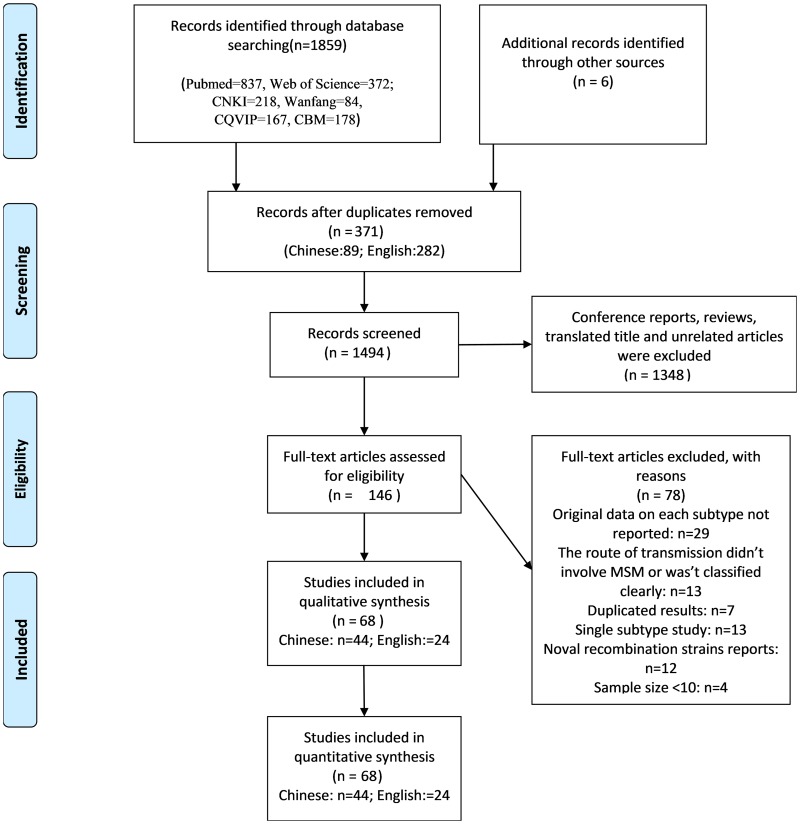
Flowchart of the study identification and selection process.

For determining the prevalence of different HIV-1 subtypes among the population of MSM in China, we found 68 independent studies that contained relevant data from 1865 records. The flow diagram in Figure 1 shows the identification process. A total of 1859 records were searched using different databases (PubMed = 837, Web of Science = 372; CNKI = 218, Wanfang = 84, CQVIP = 167, CBM = 178), and six records were searched using reference list tracing. After excluding 371 duplicates records, 1494 titles and abstracts were scanned and 1348 unrelated articles were removed. A total of 146 full-text articles were read, and 68 studies containing relevant data were included in the final study.

A total of 9132 successfully genotyped samples were collected among the 68 independent studies (44 Chinese studies and 24 English studies), involving 17 different provinces or cities (Liaoning, Jilin, Heilongjiang, Beijing, Tianjin, Hebei, Shaanxi, Anhui, Henan, Jiangsu, Zhejiang, Fujian, Guangdong, Guangxi, Yunnan). Studies were published from 2007 to 2017, and the study period was between 1999 and 2016. The patients sample size of the selected studies ranged from 11 to 1239 (median = 60.5). Detailed information is shown in Supplementary Table S1.

Generally, nested PCR, reverse transcription-PCR (RT-PCR), or nested RT-PCR were used to identify different HIV-1 subtypes, and *env*, *gag* and *pol* gene fragments were commonly used in amplification. HIV-1 subtypes were identified based on different gene fragments. Among them, 10 studies identified subtypes with three genes, 20 studies identified subtypes with two genes and 38 studies identified subtypes with only one of the three genes. Considering the influence of identified gene subtypes (*env*/*gag*/*pol*), included studies were categorized as follows: ‘A = only one gene’, ‘B = two genes’ and ‘C = all three genes’. The results showed that except for CRF01_AE and the URFs, there was no significant difference among these categories. Details are shown in Table S4. Therefore, the meta-analysis was executed using subtype data according to the author of each study, with no distinction made between combined identification or single identification.

Our systematic review revealed that the genetics of HIV among MSM are complex, including three subtypes (A: including A1; B: including Europe B and Thailand B; C), 12 CRFs (CRF01_AE, CRF07_BC, CRF08_BC, CRF_01B, CRF15_01B, CRF33_01B, CRF52_01B, CRF55_01B, CRF59_01B, CRF65_cpx, CRF67_01B, CRF68_01B), 4URFs (AE/BC, AE/B, AE/B/C, 01_AE/C) and some unknown strains. Because some CRFs have a very low frequency, we merged these with CRF_01B (constitute with CRF15_01B, CRF33_01B, CRF52_01B, CRF55_01B, CRF59_01B, CRF65_cpx, CRF67_01B and CRF68_01B); however, CRF55_01B was the predominant strain among these. The main HIV-1 subtypes among included studies were CRF01_AE (ranged from 24.44% to 90%), CRF07_BC (ranged from 1.92% to 54.55%), subtype B and/or B′ (ranged from 1.09% to 84.44%), CRF_01B (ranged from 0.49% to 50.00%), and also some URFs (ranging from 0.56% to 20.69%) and other subtypes.

### Meta-analysis of HIV-1 subtype prevalence

Pooled estimates for the different subtypes were computed via meta-analysis methods using R software. The overall proportions were as follows: 57.36% (95% CI 53.76–60.92) for CRF01_AE, 19.47% (95% CI 16.37–22.75) for CRF07_BC, 17.37% (95% CI 14.13–20.86) for B/B′, 2.67% (95% CI 0.98–4.97) for CRF08_BC, 4.46% (95% CI 2.40–7.04) for CRF_01B, 1.20% (95% CI 0.35–2.43) for C and 4.02% (95% CI 2.66–5.61) for URFs. Heterogeneity was found to be statistically significant in all subtypes; *I*^2^ ranged from 69.1% to 94.2%.

#### Over time tendency

**Fig. 2.**
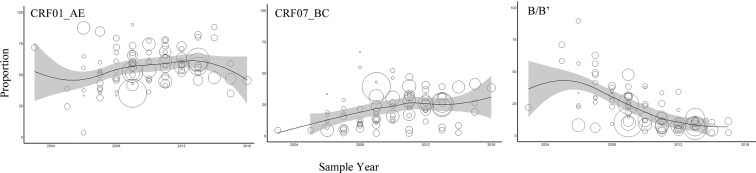
Temporal trends in the yearly proportion of different HIV-1 subtypes among MSM in China. The *x*-axis represents the midpoint of the year in which study samples were collected and analysed. The diameter of each bubble is proportional to the sample size of each study. The fitted line was plotted using locally weighted regression.

HIV-1 prevalence and epidemic trends have changed among Chinese MSM from 2000 to 2016 (Figure 2 and Table S2). For subtype CRF01_AE, the prevalence rate increased rapidly from 2006 to 2009, and then increased with slower growth from 2009 to 2013; interestingly, the prevalence decreased after 2013. Subtype CRF07_BC has remained in an increasing trend. Subtype B decreased rapidly during all of the collected study periods. The prevalence of other subtypes was all at a very low level, so temporal trends could not be clearly described; for this, long-term molecular monitoring is necessary.

#### Prevalence of HIV-1 subtypes in different regions

**Fig. 3.**
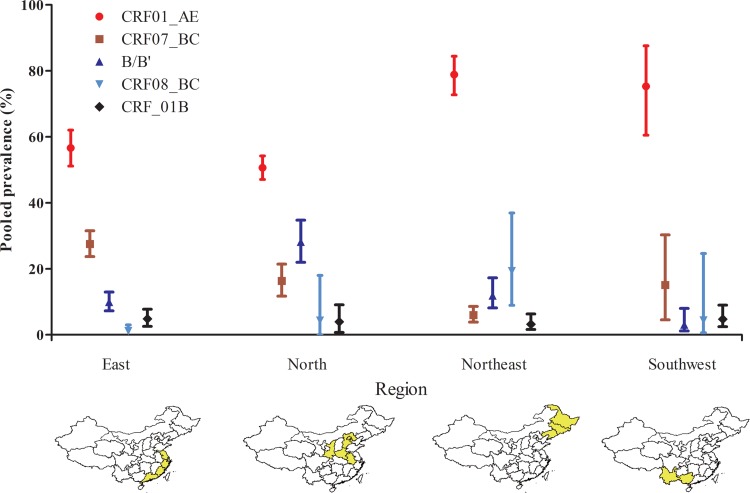
Estimated prevalence of different HIV-1 subtypes among MSM across the four regions in China during 2000–2016.

We found that the epidemic tendency among different regions has also varied (Fig. 3 and Table S3). Subtype CRF01_AE remains the predominant subtype among MSM in China with prevalence more than 50% in all regions of the country, according to our findings. In addition, CRF07_BC, CRF08_BC and B subtypes are also present. CRF07_BC has a higher prevalence in the East region (27.53%) than in the North (16.30%) and South-west (15.02%) areas, with the lowest prevalence in North-east China (5.73%). B/B′ is higher in the North (28.15%) than in other regions (East: 9.95%, North-east: 10.83%, and South-west: 3.05%). The prevalence of CRF08_BC is less than 5% in East (1.16%), North (4.33%) and South-east (4.64%), but more than 5% in the North-east China (19.35%).

**Fig. 4.**
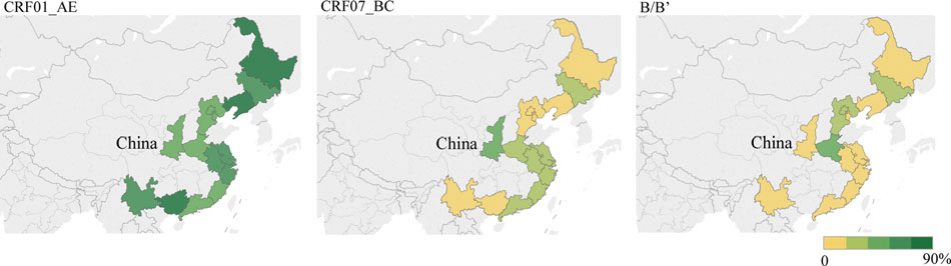
The pooled rate (%) of CRF01_AE, CRF07_BC and B/B′ among MSM in different provinces.

**Fig. 5.**
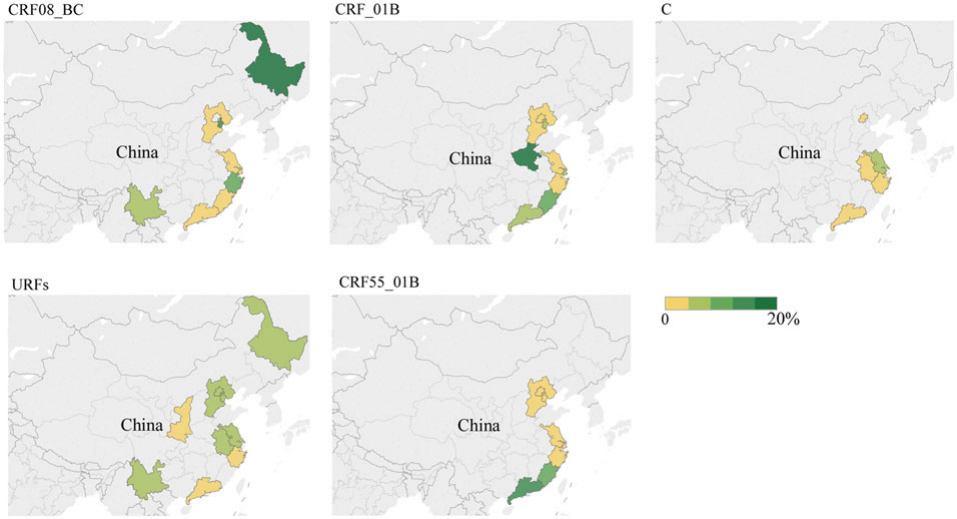
The pooled rate (%) of CRF08_BC, CRF_01B, C, URFs and CRF55_01B among MSM in different provinces.

In addition, we pooled the prevalence of subtypes by province, and the situation also varied. The prevalence of subtype B is highest in Henan (37.19%), Beijing (32.52%) and Hebei (32.29%). The prevalence of subtype CRF01_AE is highest in Guangxi (90.00%) and Yunnan (71.39%), Liaoning (81.16%) and Heilongjiang (79.17%) provinces. The prevalence of CRF07_BC is highest in Shaanxi (42.90%), followed by provinces along the South-east coast such as Guangdong (32.21%), Fujian (29.91%), Zhejiang (28.91%) and Shanghai (26.54%). Specifically, similar to Guangdong (12.22%, 95% CI 10.34–13.17) and Fujian (8.65%, 95% CI 4.98–13.17), we observed that CRF55_01B has increased in the previous 5 years, especially in South and East China. Details are shown in Figures 4 and 5.

#### Publication bias and sensitivity analysis

To detect publication bias in the included studies, we created an inverted funnel diagram including the different subtypes, and we observed asymmetry in some subtypes (Fig. S1). The Egger's test showed that publication bias may exist. Because of possible bias, further sensitivity analysis was done to estimate the stability of this analysis. Results showed that no single study influenced the overall estimated prevalence rate. After omitting one of the included studies, the estimated rate changed by less than 0.8% (Figs. S2 and S3). In addition, there was no difference in the estimated prevalence of different subtypes between Chinese and English language studies.

## Discussion

The prevalence of different HIV-1 subtypes among MSM has experienced a tremendous change in China. Our research provided a quantitative summary for these changes. In total, 68 eligible studies published from 2007 to 2017 were included. The study period covers nearly 20 years from 1999 to 2016, and the research areas included 17 different provinces and municipalities in China. When we searched the related articles, we retrieved one similar systematic review study entitled ‘Prevalence of HIV-1 subtypes among men who have sex with men in China’ [22]. This study included 19 eligible articles and the search included articles published up to 25 December 2013. As one of the key populations in China, the HIV-1 epidemic among MSM has been in an increasing period, with published studies reporting increasingly higher seroprevalence of HIV-1 in this population. In addition, the prevalence of HIV-1 subtypes has changed recently. Compared with above mentioned systematic review our analysis enrolled nearly four times the eligible articles included in that study, including all studies in that systematic review as well as an additional 49 studies published from 2013 to 2017. The pooled estimate rates for the different HIV-1 subtypes among MSM exhibited some changes compared with the rates from 4 years earlier; therefore, up-to-date information and extended monitoring is critical.

There were four HIV-1 subtypes, 12 CRFs and four URFs detected in the studies included in the present analysis. Another nationwide review summarised that eight subtypes and 21 CRFs have been detected in China. Compared with that review, we found two new CRFs detected among MSM, including CRF52_01B [29, 30] reported in Shanghai, and CRF33_01B [25] reported in Shenzhen. These subtypes identified among MSM in China indicate the complexity of HIV-1 genetics in this population and indicate connections among MSM from different areas or even other countries.

CRF01_AE is the main epidemic HIV-1 strain among MSM; we estimated that more than half of MSM are infected with CRF01_AE (57.36%, 95% CI 53.76–60.92). Initially detected among commercial sex workers and IDUs between 1994 and 1996 in Yunnan and Guangxi provinces [17], CRF01_AE has now been widely disseminated throughout China via sexual transmission [31]. As the most dominant HIV-1 strain in the country, CRF01_AE has largely contributed to the epidemic in China. Previous meta-analysis have showed that the proportion of CRF01_AE was 34.4% [31] in HIV-1 positive people and 46.3% [32] in individuals with heterosexual transmission (HST), which are both lower than the proportion among MSM. There are many reasons for the epidemic situation. Previous studies have confirmed that receptive anal intercourse leads to a higher probability of HIV-1 virus transmission and sexual-network-level risks can also be important drivers of HIV-1 viral spread [33]. Phylodynamics of CRF01_AE indicate that there are five main epidemic clusters in mainland China and two large clusters with high-level prevalence found among MSM [34]. Meanwhile, the genetic transmission network of CRF01_AE reveals that MSM have higher transmission rates than individuals who mostly had HST [35].

In this study, we found that the total estimated rate of CRF01_AE among MSM was similar to that in a systematic review by Zhang *et al*. [22] (57.36% *vs.* 53.46%). In addition, the difference in the pooled rate of CRF01_AE calculated for the same time and place was not significant between the two studies: study time was earlier than 2007 (45.16% *vs.* 42.33%), 2008–2010 (57.62% *vs.* 59.47%) and 2011–2013 (58.82% *vs.* 59.47%), sampling area in Beijing (48.97% *vs.* 50.71%) and sampling area Henan (44.18% *vs.* 43.85%). One study suggested that the molecular epidemiology trend of HIV-1 is getting more complex [16]. In our study, the pooled rate of CRF_01B was ranked fourth, followed by CRF01_AE, CRF07_BC and B/B′. These results are unusual compared with those of previous systematic reviews [22, 23, 31]. Furthermore, we found a downward trend in the prevalence of CRF01_AE during the previous 4 years (Fig. 2). URFs among MSM in China have shown a gradual upward trend. URFs, which nearly always consisted of a recombination of CRF01_AE with subtype B or CRF07_BC, were the most common HIV-1 subtypes identified in the MSM population [34, 36]. Therefore, we speculate that CRF01_AE is still circulating among MSM in China. Nevertheless, more strains have now recombined to form URFs. This upward trend in recombinant subtypes of HIV-1 among MSM in China like URFs should alarm the Chinese CDC and other relevant departments.

CRF07_BC is one of the other most dominant HIV-1 strains in China and is believed to have originated in Yunnan province in the 1980s [17], spreading along drug trafficking routes by IDUs to Sichuan, Guangxi and Xinjiang provinces [37] in Southwest and North China. Phylodynamic analysis has identified three CRF07_BC clusters and two rapid spreading waves of CRF07_BC infection in China. Two clusters mainly comprised sequence from IDUs and contributed to the first spreading waves, in addition, another cluster comprised sequence from MSM and caused the second spreading waves in about 2000 [38]. Besides, molecular epidemiologic investigation among young (aged 16–25 years) MSM have identified that a new cluster entered China in 2004, and the evidence implied that MSM either had more sex with IDUs or more MSM use drugs around the year 2000 [24]. The result of our study showed CRF07_BC increased from 2004 to 2010, and then experienced slower growth, which is consistent with the evidence. A laboratory study found that in some MSM, CRF07_BC strains may significantly improve *in vitro* infectivity [39]. A clinical study found that after 18 months of treatment, total HIV DNA decreases more markedly in patients infected with CRF01_AE than with subtype B or CRF07_BC [40]. Therefore, higher prevalence in young MSM, stronger infectivity, and higher viral load after treatment may have led to this growing epidemic. CRF08_BC was also initially found in Yunnan and Guangxi provinces among IDUs, similar to CRF07_BC, but it has rarely been reported in MSM. Compared with the previous systematic review, total pooled estimate of CRF08_BC (5.9% *vs.* 2.7%) was lower in our analysis, but we found no obvious trend in recent years, which is probably associated with sample size.

The result of our study showed that the prevalence of subtype B (17.37%, 95% CI 14.13–20.86) declined rapidly in MSM; the estimated percentage of subtype B was 10.88% lower than the percentage 4 years earlier (28.25%, 95% CI 18.10–39.66). Phylogenetic analysis revealed that there were four types of B strains (named according to the origin, B′ (Thai-B), BJ-B (Beijing-B), Pan-B (Pandemic-B) and TW-B (Taiwan-B)), circulating in China respectively [41]. B/B′ was originally detected among IDUs in Yunnan during the 1990s and was then introduced into central China (Henan, Hebei and Anhui provinces). Outbreaks among former plasma donors in those areas subsequently spread nationwide [42]. Our result showed that Henan (37.19%), Hebei (32.29) and Beijing (32.52%) had a high-level epidemic of subtype B, which is supported by the above evidence. Although the prevalence of subtype B was in a declining trend, B/B′ is still the third most prevalent strain and is responsible for 17.37% of HIV infection among MSM in the whole country. Besides, new CRFs and URFs always consist of by B/B′ and other subtypes like CRF_01B [43], CRF_BC [44], CRF67_cpx [45] and among others, which may still impact the epidemic in China.

CRF55_01B has been identified among MSM in Shenzhen [46]. We observed that CRF55_01B has increased in the past 5 years, especially in South and East China, such as in Guangdong (12.22%, 95% CI 10.34–13.17) and Fujian (8.65%, 95% CI 4.98–13.17). A large-scale molecular epidemiologic survey had similar results, with the prevalence of CRF55_01B at 12.5% in Guangdong and 7.4% in Hunan; however, no CRF55_01B has been found in northern China [47]. Our study showed that CRF55_01B has also been detected in Beijing (0.54%, 95% CI 0.00–1.84) and Tianjin City (0.99%, 95% CI 0.00–4.20) in Northern China. As the economic and cultural centre of China, this epidemic suggested that CRF55_01B may spread throughout northern China through large cities.

To summarise the findings above, there are some suggestions for future preventions efforts targeting HIV-1 epidemics among MSM. As CRF01_AE are the main subtypes of HIV-1 epidemic among MSM in China, HIV positive patients who are infected with CRF01_AE or other URFs subtypes require greater attention and effective intervention. Some previous studies focusing on HIV transmission networks have indicated that CRF01_AE subtypes of HIV-1 always produce larger clustered networks [48], facilitating HIV-1 transmission among groups of MSM. Further, some studies have showed that many newly detected HIV virus strains are more likely to recombine with CRF01_AE subtypes [34, 35], so timely HIV screening and treatment are urgently needed for MSM in China. In addition, previous research has shown that the migrant MSM had higher rates of recreational drug use, multiple sexual partners, participation in commercial sex and other characteristics [49–52] these factors put these individuals at higher risk for HIV acquisition. Thus, there is an urgent need for HIV-related behavioural interventions for migrant MSM, especially in large cities. There are more opportunities for work, convenient transportation and prosperous economies in large cities of China, which attract great numbers of people who work and live in these cities, including MSM. Furthermore, different subtypes may have differing characteristics. For example, CRF01_AE is associated with low CD4+T cell count and a higher proportion of CXCR4 co-receptor in MSM [53]. These different features may influence treatment and vaccine development, so drugs and vaccines targeting different subtypes are critically needed.

Considering the methods of subtype identification according to different genetic regions among the studies included here, we also performed a subgroup analysis of the different strategies. (Table S4) and found that there was no significant difference among most subtypes (except for CRF01_AE and URFs). The previously mentioned meta-analysis among MSM found differences in the pooled estimated rate among different gene regions (env/gag/pol) [22]. Another study regarding HST undertook in a similar analysis, and the result also showed a difference between ‘only one group’ and ‘two or more groups’ [32]. A study by Chen's et al. reported that subtypes CRF01_AE and CRF15_01B can only be differentiated by the *env* region [54]. Deng et al. established a phylogenetic tree established by using same sample but a different gene region. The bootstrap value in the *env*-tree was higher than that in the pol-tree [55], suggesting that the HIV-1 gene region can influence subtypes identification; therefore, near full-length genome sequencing is still needed to confirm additional CRFs and URFs.

Although this meta-analysis was carried out using a comprehensive database search and following strict inclusion and exclusion criteria, there were still some limitations. First, the frequency or percentage of subtypes was not reported for different study populations, and it was not possible to disaggregate data of MSM. Thus, some studies were excluded. Second, half of the studies identified subtypes by a single HIV-1 genetic region, which may mistyping of some HIV-1 subtypes, thus influencing the estimated prevalence. Third, there was large heterogeneity among the included studies, which still existed in subgroup analysis; this could be associated with the small sample size in some studies, as well as different sample source and study periods. In addition, sensitivity analysis showed that our results were stable; however, publications bias existed, which could be related to the large heterogeneity among the different studies.

In conclusion, our meta-analysis offered an exhaustive overview of temporal trend and geographic changes in HIV-1 subtypes among MSM in China. The results showed that CRF01_AE, CRF07_BC and B were still predominant HIV-1 strains circulating among MSM, with different spatiotemporal trends. We initially observed CRF01_AE had followed a downward trend in the past 4 years and that CRF55_01B has increased in Eastern China. Recently, HIV-1 strains have become more complex, with more unusual CRFs and URFs being detected. Large cities may be sites from which new recombinant strains originate and may become centres of viral spread. For control and prevention of the HIV-1 epidemic among the important MSM population long-term molecular monitoring is necessary and meaningful.

## Supplementary material

For supplementary material accompanying this paper visit https://doi.org/10.1017/S0950268818003400. click here to view supplementary material
