# Supplementary material for: The prevalence, temporal trends, and geographical distribution of HIV-1 subtypes among men who have sex with men in China: A systematic review and meta-analysis
Source: Epidemiol Infect. 2019 Feb 19;147:e83. doi: 10.1017/S0950268818003400 (PMC6518548; doi:10.1017/S0950268818003400)
Supplement: Supplementary file 1 [file S0950268818003400sup001.zip › S0950268818003400sup001/FigureS3.docx]

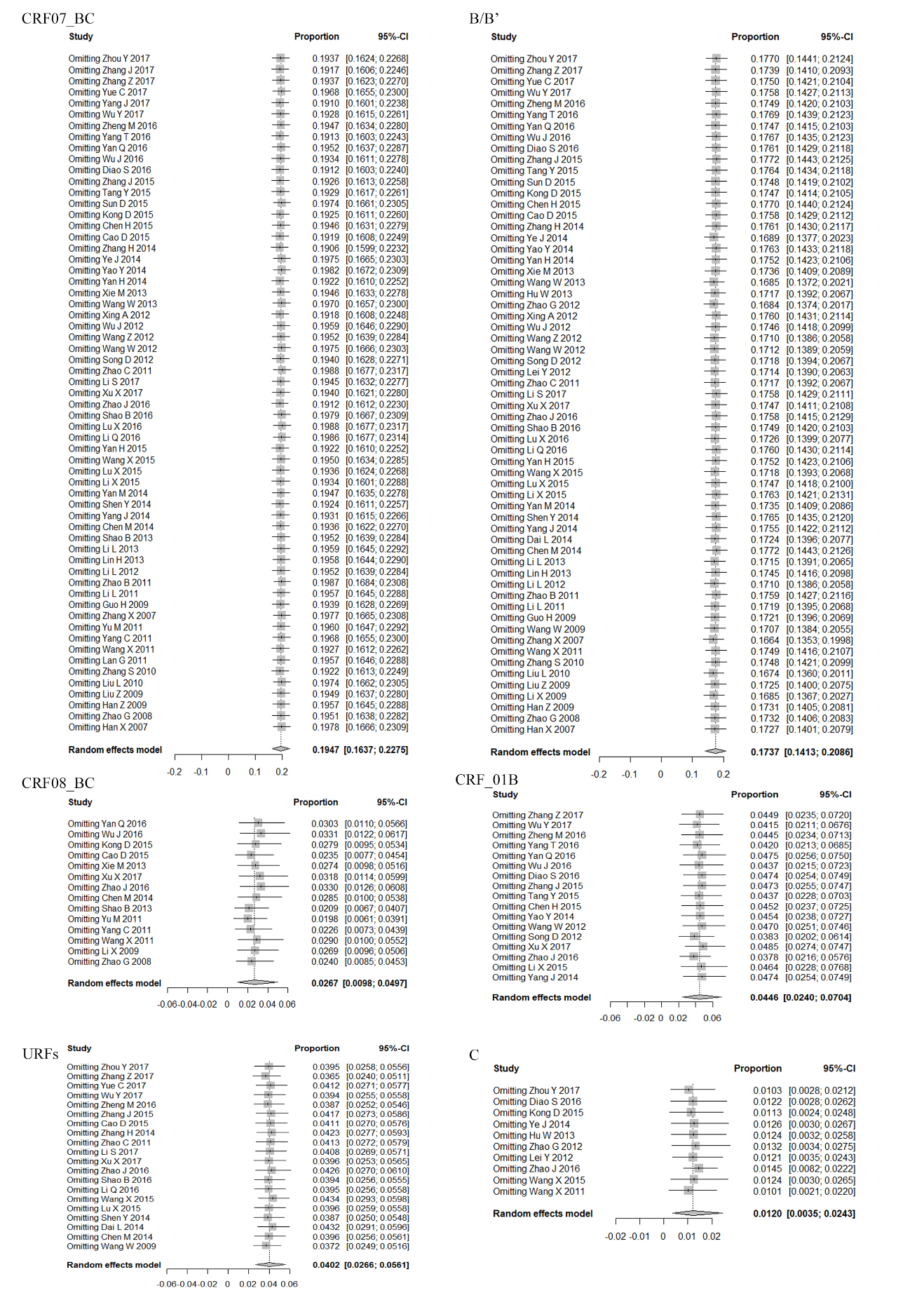


Figure S3: The forest plot of sensitivity analysis of the proportion of different HIV-1 subtypes (CRF07_BC, B/B’, CRF08_BC, CRF_01B, C, URFs).
