# Supplementary material for: The prevalence, temporal trends, and geographical distribution of HIV-1 subtypes among men who have sex with men in China: A systematic review and meta-analysis
Source: Epidemiol Infect. 2019 Feb 19;147:e83. doi: 10.1017/S0950268818003400 (PMC6518548; doi:10.1017/S0950268818003400)
Supplement: Supplementary file 1 [file S0950268818003400sup001.zip › S0950268818003400sup001/TableS1.docx]

Table S1. Characteristics of included studies and the prevalence of different HIV-1 subtypes among MSM in China

| First author, Published year | Location (city, province) | Study time | Age | Gene Amplification Region | Samples | Prevalence of HIV-1 subtypes | | | | | | |
| --- | --- | --- | --- | --- | --- | --- | --- | --- | --- | --- | --- | --- |
|  |  |  |  |  |  | CRF01_AE | CRF07_BC | B/B' | CRF08_BC | CRF_01B | C | URFs |
| Zhou Y, 2017[^1^](#_ENREF_1) | Jiangsu | 2015 | mean age 22 (16-25) | pol | 46 | 58.70% | 26.09% | 2.17% | -- | -- | 6.52% | 6.52% |
| Zhang J, 2017[^2^](#_ENREF_2) | Yiwu, Zhejiang | 2016 | mean age 39.1±13.4 | pol | 86 | 45.35% | 38.37% |  | -- | -- | -- | -- |
| Zhang Z, 2017[^3^](#_ENREF_3) | Beijing | 2010-2012 | mean age 31.8(18-49) | pol, gag, env | 96 | 42.71% | 25.00% | 16.67% | -- | 4.17% | -- | 11.46% |
| Yue C, 2017[^4^](#_ENREF_4) | Harbin, Heilongjiang | 2014 | mean age 36.2(20-55) | gag | 49 | 79.59% | 8.16% | 5.20% | -- | -- | -- | 2.04% |
| Yang J, 2017[^5^](#_ENREF_5) | Zhejiang | 2015 | mean age 35.8±13.9 | pol | 150 | 46.67% | 41.33% |  | -- | -- | -- | -- |
| Wu Y, 2017[^6^](#_ENREF_6) | Guangdong | 2013 | mean age 28(17-47) | pol, gag, env | 123 | 45.53% | 30.08% | 10.13% | -- | 10.57% | -- | 5.69% |
| Zheng M, 2016[^7^](#_ENREF_7) | Tianjin | 2015 | mean age  33±10(12-58) | pol, env | 63 | 34.92% | 19.05% | 7.11% | -- | 4.76% | -- | 7.94% |
| Yang T, 2016[^8^](#_ENREF_8) | Fujian | 2012-2015 | mean age 29.2±8.6 | pol, gag | 185 | 47.57% | 38.92% | 8.32% | -- | 9.19% | -- | -- |
| Yan Q, 2016[^9^](#_ENREF_9) | Huaian, Jiangsu | 2005-2016 | NR | env | 173 | 68.21% | 16.76% | 23.29% | 0.58% | 1.16% | -- | -- |
| Wu J, 2016[^10^](#_ENREF_10) | Shanghai | 2013 | mean age 32.1±10.3(17-80) | pol | 645 | 62.17% | 25.27% | 37.74% | 0.16% | 6.67% | -- | -- |
| Diao S, 2016[^11^](#_ENREF_11) | Guangzhou, Guangdong | 2011 | mean age 30.6±1.2 | gag, env | 220 | 44.55% | 39.55% | 16.27% | -- | 1.36% | 1.36% | -- |
| Zhang J, 2015[^12^](#_ENREF_12) | Zhejiang | 2011 | NR | pol | 101 | 62.38% | 31.68% | 3.97% | -- | 0.99% | -- | 1.98% |
| Tang Y, 2015[^13^](#_ENREF_13) | Shanghai | 2011-2014 | mean age 31.06±9.86(20-64) | pol | 62 | 58.06% | 30.65% | 3.84% | -- | 6.45% | -- | -- |
| Sun D, 2015[^14^](#_ENREF_14) | Harbin, Heilongjiang | 2012-2014 | mean age 30.2±9.95 | gag | 61 | 81.97% | 6.56% | 7.48% | -- | -- | -- | -- |
| Kong D, 2015[^15^](#_ENREF_15) | Shenzhen, Guangdong | 2007-2010 | NR | gag, env | 258 | 51.16% | 31.01% | 35.57% | 2.33% | -- | 1.94% | -- |
| Chen H, 2015[^16^](#_ENREF_16) | Wuxi, Jiangsu | 2012-2013 | age 18-77 | env | 110 | 72.73% | 20.00% | 4.64% | -- | 3.64% | -- | -- |
| Cao D, 2015[^17^](#_ENREF_17) | Shaoxing, Zhejiang | 2013-2014 | mean age 32.22 | pol, gag | 46 | 52.17% | 39.13% | 3.52% | 8.70% | -- | -- | 2.17% |
| Zhang H, 2014[^18^](#_ENREF_18) | Xi'an, Shaanxi | 2010-2012 | mean age 32(14-61) | gag, env | 168 | 47.02% | 44.05% | 12.14% | -- | -- | -- | 1.79% |
| Ye J, 2014[^19^](#_ENREF_19) | Beijing | 2006-2010 | mean age 30.4(19-62) | pol | 189 | 46.56% | 7.94% | 84.44% | -- | -- | 1.06% | -- |
| Yao Y, 2014[^20^](#_ENREF_20) | Zhejiang | 2004-2011 | age 19-49 | pol, gag | 117 | 84.62% | 5.13% | 7.98% | -- | 3.42% | -- | -- |
| Yan H, 2014[^21^](#_ENREF_21) | Shanghai | 2012-2013 | NR | pol | 52 | 53.85% | 36.54% | 5.62% | -- | -- | -- | -- |
| Xie M, 2013[^22^](#_ENREF_22) | Fujian | 2007-2010 | mean age  27±6.9(16-50) | pol | 50 | 60.00% | 20.00% | 9.00% | 2.00% | -- | -- | -- |
| Wang W, 2013[^23^](#_ENREF_23) | Beijing | 2005-2009 | NR | env | 130 | 41.54% | 9.23% | 63.46% | -- | -- | -- | -- |
| Hu W, 2013[^24^](#_ENREF_24) | Beijing | 2007-2009 | mean age 35.02±9.15(21-60) | gag, env | 95 | 58.95% | -- | 28.47% | -- | -- | 1.05% | -- |
| Zhao G, 2012[^25^](#_ENREF_25) | Shenzhen, Guangdong | 2008-2010 | mean age 30.8 | pol, gag, env | 164 | 41.46% | -- | 78.56% | -- | -- | 0.61% | -- |
| Xing A, 2012[^26^](#_ENREF_26) | Shaanxi | 2011 | mean age 33(21-69) | pol, gag, env | 51 | 54.90% | 39.22% | 3.88% | -- | -- | -- | -- |
| Wu J, 2012[^27^](#_ENREF_27) | Shang Hai | 2010 | age 20-60 | pol | 26 | 76.92% | 11.54% | 3.54% | -- | -- | -- | -- |
| Wang Z, 2012[^28^](#_ENREF_28) | Zhengzhou, Henan | 2010 | mean age 33.2(21-59) | pol, gag, env | 31 | 45.16% | 16.13% | 12.71% | -- | -- | -- | -- |
| Wang W, 2012[^29^](#_ENREF_29) | Beijing | 2005-2011 | NR | gag | 183 | 48.63% | 7.65% | 58.69% | -- | 1.64% | -- | -- |
| Song D, 2012[^30^](#_ENREF_30) | Zhengzhou, Henan | 2011 | mean age 29.25(21-58) | gag | 24 | 41.67% | 25.00% | 8.33% | -- | -- | -- | -- |
| Song D, 2012[^31^](#_ENREF_31) | Zhengzhou, Henan | 2011 | mean age 29.25(21-58) | env | 18 | 22.22% | 22.22% | 5.56% | -- | 50.00% | -- | -- |
| Lei Y, 2012[^32^](#_ENREF_32) | Hefei, Anhui | 2011-2012 | mean age 31(17-58) | pol | 35 | 57.14% |  | 12.29% | -- | -- | 2.86% | -- |
| Zhao C, 2011[^33^](#_ENREF_33) | Hebei | 2009 | NR | gag, env | 52 | 65.38% | 1.92% | 16.77% | -- | -- | -- | 1.92% |
| Li S, 2017[^34^](#_ENREF_34) | Zhenjiang, Jiangsu | 2012-2013 | NR | gag, env | 34 | 70.59% | 20.59% | 2.88% | -- | -- | -- | 2.94% |
| Lu X, 2017[^35^](#_ENREF_35) | Hebei | 2013 | NR | pol, gag | 411 | 56.93% | 22.38% | 58.11% | 0.49% | 0.49% | -- | 5.35% |
| Zhao J, 2016[^36^](#_ENREF_36) | Shenzhen, Guangdong | 2005-2012 | mean age 34.8(20-79) | pol | 996 | 35.14% | 39.06% | 10.14% | 0.10% | 12.85% | 0.10% | 2.61% |
| Shao B, 2016[^37^](#_ENREF_37) | Harbin | 2009-2013 | NR | gag | 63 | 77.78% | 4.76% | 7.11% | -- | -- | -- | 6.35% |
| Lu X, 2016[^38^](#_ENREF_38) | Hebei | 2011 | NR | gag, env | 52 | 73.08% | 1.92% | 13.00% | -- | -- | -- | -- |
| Li Q, 2016[^39^](#_ENREF_39) | Harbin, Heilongjiang | 2013-2014 | mean age 33.8 | gag | 87 | 82.76% | 3.45% | 6.90% | -- | -- | -- | 5.75% |
| Yan H, 2015[^40^](#_ENREF_40) | Shanghai | 2012-2013 | NR | pol | 52 | 53.85% | 36.54% | 5.62% | -- | -- | -- | -- |
| Wang X, 2015[^41^](#_ENREF_41) | Beijing | 2007-2010 | mean age 32(19-66) | pol | 179 | 51.96% | 17.88% | 51.49% | -- | -- | 1.12% | 0.56% |
| Lu X, 2015[^42^](#_ENREF_42) | Hebei | 2013 | mean age 32.44±9.63(18-56) | pol, gag, env | 50 | 56.00% | 26.00% | 6.00% | -- | -- | -- | 6.00% |
| Li X, 2015[^43^](#_ENREF_43) | Shanghai | 2009-2013 | mean age 30.6±9.55(18-79) | pol | 1263 | 63.44% | 25.02% | 8.23% | -- | 3.31% | -- | -- |
| Yan M, 2014[^44^](#_ENREF_44) | Jilin | 2012 | mean age 38.9±8.1(23-57) | pol, gag | 15 | 60.00% | 20.00% | 3.00% | -- | -- | -- | -- |
| Shen Y, 2014[^45^](#_ENREF_45) | Anhui | 2011 | mean age 31.6(18-68) | pol | 133 | 55.64% | 32.33% | 7.26% | -- | -- | -- | 6.77% |
| Yang J, 2014[^46^](#_ENREF_46) | Beijing | 2011-2013 | mean age 30.3(17-64) | pol | 223 | 60.54% | 27.80% | 22.87% | -- | 1.35% | -- | -- |
| Dai L, 2014[^47^](#_ENREF_47) | Beijing | 2008-2011 | NR | pol | 536 | 52.05% | -- | 24.81% | -- | -- | -- | 1.31% |
| Chen M, 2014[^48^](#_ENREF_48) | Kunming, Yunnan | 2010-2012 | medium age 26(17-70) | pol, gag, env | 131 | 64.89% | 25.19% | 4.05% | 1.53% | -- | -- | 5.34% |
| Shao B, 2013[^49^](#_ENREF_49) | Harbin, Heilongjiang | 2011 | age 17-70 | env | 31 | 64.52% | 16.13% | -- | 19.35% | -- | -- | -- |
| Li L, 2013[^50^](#_ENREF_50) | Beijing | 2007-2010 | mean age 33.2(23-57) | pol, gag, env | 91 | 56.04% | 13.19% | 28.77% | -- | -- | -- | -- |
| Lin H, 2013[^51^](#_ENREF_51) | Taizhou, Zhejiang | 2008-2010 | NR | gag, env | 60 | 73.33% | 13.33% | 8.33% | -- | -- | -- | -- |
| Li L, 2012[^52^](#_ENREF_52) | Henan | 2010 | mean age 33.2(21-59) | pol, gag, env | 31 | 45.16% | 16.13% | 12.71% | -- | -- | -- | -- |
| Zhao B, 2011[^53^](#_ENREF_53) | Liaoning | 2003-2009 | mean age 36(18-71) | pol | 201 | 87.56% | 4.48% | 16.96% | -- | -- | -- | -- |
| Li L, 2011[^54^](#_ENREF_54) | Shijiazhuang, Hebei | 2008 | mean age 33.2(23-57) | env | 17 | 52.94% | 11.76% | 6.29% | -- | -- | -- | -- |
| Guo H, 2009[^55^](#_ENREF_55) | Jiangsu | 2007 | NR | gag, env | 14 | 35.71% | 28.57% | 5.71% | -- | -- | -- | -- |
| Wang W, 2009[^56^](#_ENREF_56) | Beijing | 2007 | mean age 30.8±8（19-56） | pol, gag, env | 29 | 37.93% | -- | 12.38% | -- | -- | -- | 20.69% |
| Zhang X, 2007[^57^](#_ENREF_57) | Beijing | 2005-2006 | mean age 31(19-54) | pol, gag, env | 45 | 24.44% | 4.44% | 32.11% | -- | -- | -- | -- |
| Yu M, 2011[^58^](#_ENREF_58) | Tianjin | 2008 | mean age 28.6±8(18-53) | pol, gag | 50 | 72.00% | 12.00% | -- | 16.00% | -- | -- | -- |
| Yang C, 2011[^59^](#_ENREF_59) | Yunnan | 2008 | NR | env | 48 | 79.17% | 8.33% | -- | 10.42% | -- | -- | -- |
| Wang X, 2011[^60^](#_ENREF_60) | Shenzhen, Guangdong | 2010 | NR | gag, env | 249 | 53.01% | 30.12% | 31.45% | 1.61% | -- | 2.81% | -- |
| Lan G, 2011[^61^](#_ENREF_61) | Nanning, Guangxi | 2009 | NR | gag | 10 | 90.00% | 10.00% | -- | -- | -- | -- | -- |
| Zhang S, 2010[^62^](#_ENREF_62) | Ningbo, Zhejiang | 2008-2009 | mean age 30(21-40) | env | 11 | 36.36% | 54.55% | 1.09% | -- | -- | -- | -- |
| Liu L, 2010[^63^](#_ENREF_63) | Shijiazhuang, Hebei | 2006-2008 | mean age 26(18-50) | env | 51 | 31.37% | 5.88% | 32.75% | -- | -- | -- | -- |
| Liu Z, 2009[^64^](#_ENREF_64) | Beijing | 2007-2008 | NR | gag, env | 17 | 52.94% | 17.65% | 5.41% | -- | -- | -- | -- |
| Li X, 2009[^65^](#_ENREF_65) | Shijiazhuang, Hebei | 2004-2006 | mean age 28.8(18-42) | env | 36 | 38.89% | -- | 21.33% | 2.78% | -- | -- | -- |
| Han Z, 2009[^66^](#_ENREF_66) | Guangzhou, Guangdong | 2006-2007 | mean age 31.84±7.54(19-48) | gag, env | 17 | 64.71% | 11.76% | 4.53% | -- | -- | -- | -- |
| Zhao G, 2008[^67^](#_ENREF_67) | Shenzhen, Guangdong | 2006 | mean age 27.1(19-45) | env | 18 | 55.56% | 16.67% | 4.22% | 16.67% | -- | -- | -- |
| Han X, 2007[^68^](#_ENREF_68) | Liaoning | 1999-2007 | mean age 36(18-70) | pol | 46 | 71.74% | 4.35% | 11.91% | -- | -- | -- | -- |

NR: not reported.

Reference:

1. Ying Z, Xiaoqin X, Jing L, et al. A survey of HIV drug resistance threshold in Jiangsu Province in 2015. *Acta Universitatis Medicinalis Nanjing（Natural Science）.* 2017;37(2).

2. Jiafeng Z, Jiaming Y, Qin F, et al. Analysis on HIV-1 subtypes and transmission clusters in newly reported HIV/AIDS cases in Yiwu, Zhejiang Province, 2016. *Chinese Journal of Epidemiology.* 2017.

3. Zhimin Z, Wei X, Yuefang Z, et al. Dynamics of HIV-1 subtypes among men who have sex with men during acute infection from 2010 to 2012 in Beijing area. *CHINESE Journal of AIDS & STD.* 2017(05):382-386.

4. Chao Y, Gang J, Haining L, et al. Genotype analysis of HIV-1 gag genes from cases diagnosed as men who have sex with men in Harbin. *Chinese Journal of Experimental And Clinical Infectious Diseases.* 2017;11(1):27-31.

5. Jiezhe Y, Wanjun C, Wenjun Z, et al. Molecular epidemiology and transmission of HIV-1 infection in Zhejiang province, 2015. *Chinese Journal of Epidemiology.* 2017;38(11):1551-1556.

6. Yue W. *Comprehensive characterization of the HIV-1 molecular epidemiology among IDUs and MSM in Guangzhou* [D], Southern Medical University; 2017.

7. Minna Z, Tielin N, Yongjun G, et al. Molecular epidemiology and transmission of HIV in Tianjin，2015. *Chinese Journal of Epidemiology.* 2016;37(8):1142-1147.

8. Tianfei Y. *Study on relation of transmission and primary drug-resistant for HIV -1 infection among MSM and heterosexual in Fujian province* [D], Fujian Medical University; 2016.

9. Qingli Y, Pengfei Y, Wei F, et al. Molecular characterization of HIV-1 infection among men who have sex with men in Huai’an. *Chinese Journal of Disease Control and Prevention.* 2016;20(12):1231-1235.

10. Wu J, Wang X, Yin H, et al. Gene mutations of human immunodeficiency virus drug resistance from men who have sex with men in Shanghai, 2013. *Chinese Journal of Infectious Diseases.* 2016;34(1):23-26.

11. Shuqin D, Hao W, Kai G, et al. Study of MSM infected with HIV-1 subtype reported in 2011 in Guangzhou. *CHINESE Journal of AIDS & STD.* 2016(09):680-682.

12. Jiafeng Z, Jiezhe Y, Xiaohong P, et al. HIV-1 subtype diversity and transmission clusters among men having sex with men who recently got HIV-1 infection, in Zhejiang province. *Chinese Journal of Epidemiology.* 2015;36(1):61-66.

13. Yan T, Qiao S, Yile X, et al. Molecular epidemiology among MSM in Pudong New Area of Shanghai city. *CHINESE Journal of AIDS & STD.* 2015(06):461-465.

14. Dongying S, Bing S, Chunlei Z, et al. Analysis on the characteristics of HIV-1 gag gene among men who have sex with men living with HIV-1 in Harbin City. *Chinese Journal of Experimental And Clinical Infectious Diseases.* 2015(01):14-18.

15. Dongfeng K, Xiaohui W, Yanmin Q. Molecular epidemiological characteristics of 528 HIV-1 infected cases in Shenzhen. *China Tropical Medicine.* 2015;0(5).

16. Hao C, Rui Y, Yanxin X, et al. A molecular epidemiological study on the human immunodeficiency virus infection in Wuxi City. *Chinese Journal of Disease Control and Prevention.* 2015(12):1195-1199.

17. Dongqing C, Tingting H, Danyan Z, et al. Genotyping of HIV-1 detected in HIV infected men who have sex with men in Shaoxing, Zhejiang. *Disease Surveliance.* 2015;30(10):841-845.

18. Hailan Z, Yongping Y, Hengxin L, et al. Molecular epidemiology related to human immunodefieieney virus type I infection in men having sex with meu in Xi’an. *Chinese Journal of Epidemiology.* 2014;35(4):421-424.

19. Jingrong Y, Wanchun Z, Xueli S, et al. Morecular Epidemiological Characteristics of HIV-1 Strains Isolated from Newly Diagnosed MSM Subjects (2006-2010) in Beijing, China. *Chinese Journal OF Virology.* 2014(02):138-142.

20. Yaping Y, Jiafeng Z, Yun X, et al. Analysis on the genetic characteristics of HIV-1 infected MSM individuals in Zhejiang province. *Zhejiang Journal of preventive Medicine.* 2014(09):880-883.

21. Huamei Y. *Research on HIV-1 infection and HIV-1 molecular epidemiology among MSM in Shanghai, China* [D], Fudan University; 2014.

22. Meirong X, Yansheng Y, Pingping Y, et al. Primary drug-resistance gene mutation to HIV-1 infected MSM in Fujian Province. *Chinese Journal of Zoonoses.* 2013(01):27-29+42.

23. Wanhai W, Liang M, Shulin J, et al. The dynamic of HIV-1 subtypes among men who had sex with men from 2005-2009. *Journal of Pathogen Biology.* 2013(06):493-496.

24. Wenjing H, Xin Z, Weihua L, et al. Analysis of HIV-1 subtypes among acute HIV-1 infectors from men who have sex with men. *Journal of Addiction Medicine.* 2013(04):236-238.

25. Guanglu Z, Wei Y, Yumao C, et al. Primary drug resistance of HIV-1 infected men who have sex with men in Shenzhen, China. *National Medical Journal of China.* 2012;92(17):1165-1169.

26. Aihua X, Xiang L, Mengyan Z, et al. Moleculo-epidemiological study on HIV type ong among MSM in Shaanxi Province. *CHINESE Journal of AIDS & STD.* 2012(11):741-745.

27. Jian W, Yile X, Laiyi K. Molecular epidemiology and drug resistance: Survey on human immunodeficiency virus type 1 in Luwan District of Shanghai. *Journal of Diagnostics Concepts and Practice.* 2012(03):278-282.

28. Zhe W, Guowei C, Guoqing S, et al. Men who have sex with men and its relationship with HIV一1 strains prevailing in the paid blood donorsfrom Zhengzhou city, Henan. *Chinese Journal of Epidemiology.* 2012;33(9):888-892.

29. Wanhai W, Liang M, Shulin J, et al. The prevalence and dynamics of human immunodeficiency virus-1 subtypes among men who have sex with men in Beijing. *Chinese Journal of Infectious Diseases.* 2012;30(11):673-676.

30. Dan S, Guoqing S, Yanmin Z, et al. Subtype and Sequence Analysis of Gag Gene of HIV-1 Among Men Who Have Sex in Zhengzhou, Henan Province. *Chinese Journal OF Virology.* 2012(04):345-350.

31. Dan S, Guoqing S, Yanmin Z, et al. Subtype analysis of gp41 gene of HIV-1 among men who have sex with men in Zhengzhou. *Chinese Journal of Preventive Medicine.* 2012;46(8):728-731.

32. Yanhua L, Zhongwang H, Hai W, et al. Primary drugs resistance of HIV-1 in MSM population of Hefei, China. *Chinese Journal of Viral Diseases.* 2012(06):50-54.

33. Cuiying Z, Guangyi B, Qiaomin L, et al. Molecular epidemiology study of HIV-1/AIDS among men who have sex with men in Hebei province. *Chinese Journal of Preventive Medicine.* 2011;45(5).

34. Li S, Xiaoxia L, GengFeng F, et al. The Epidemic of Human Immunodeficiency Virus, Hepatitis C Virus, and Syphilis Infection, and the Correlates of Sexually Transmitted Infections among Men Who Have Sex with Men in Zhenjiang, Jiangsu, China. *Japanese Journal of Infectious Diseases.* Mar 2017;70(2):171-176.

35. Xinli L, Xianjiang K, Yongjian L, et al. HIV-1 molecular epidemiology among newly diagnosed HIV-1 individuals in Hebei, a low HIV prevalence province in China. *Plos One.* Feb 2017;12(2).

36. Zhao J, Chen L, Chaillon A, et al. The dynamics of the HIV epidemic among men who have sex with men (MSM) from 2005 to 2012 in Shenzhen, China. *Scientific Reports.* Jun 2016;6.

37. Bing S, Bo S, Lijun C, et al. Molecular epidemiology is becoming complex under the dynamic HIV prevalence: The perspective from Harbin, China. *Journal of Medical Virology.* May 2016;88(5):807-814.

38. Xinli L, Cuiying Z, Wei W, et al. HIV-1 genetic diversity and its distribution characteristics among newly diagnosed HIV-1 individuals in Hebei province, China. *Aids Research and Therapy.* Jan 2016;13.

39. Qing-Hai L, Fu-Xiang W, Chao Y, et al. Molecular Genotyping of HIV-1 Strains from Newly Infected Men Who Have Sex with Men in Harbin, China. *Aids Research and Human Retroviruses.* Jun 2016;32(6):595-600.

40. Huamei Y, Yingying D, Y. WF, et al. Epidemiological and molecular characteristics of HIV infection among money boys and general men who have sex with men in Shanghai, China. *Infection Genetics and Evolution.* Apr 2015;31:135-141.

41. Xicheng W, Yasong W, Xia ML, et al. Targeting HIV Prevention Based on Molecular Epidemiology Among Deeply Sampled Subnetworks of Men Who Have Sex With Men. *Clinical Infectious Diseases.* Nov 2015;61(9):1462-1468.

42. Xinli L, Xianjiang K, Suliang C, et al. HIV-1 Genetic Diversity and Transmitted Drug Resistance Among Recently Infected Individuals at Men Who Have Sex with Men Sentinel Surveillance Points in Hebei Province, China. *Aids Research and Human Retroviruses.* Oct 2015;31(10):1038-1045.

43. Xiaoyan L, Yile X, Hua C, et al. HIV-1 Genetic Diversity and Its Impact on Baseline CD4+T Cells and Viral Loads among Recently Infected Men Who Have Sex with Men in Shanghai, China. *Plos One.* Jun 2015;10(6).

44. Ming Y, Ke Z, Juan D, et al. HIV-1 Diversity and Drug-Resistant Mutations in Infected Individuals in Changchun, China. *Plos One.* Jun 2014;9(6).

45. Yuelan S, Bin S, Jianjun W, et al. The prevalence of transmitted HIV drug resistance among MSM in Anhui province, China. *AIDS Research and Therapy.* 2014;11:19.

46. Yang J, Shuming L, Zhenpeng L, et al. HIV-1 transmitted drug resistance-associated mutations and mutation co-variation in HIV-1 treatment-naive MSM from 2011 to 2013 in Beijing, China. *Bmc Infectious Diseases.* Dec 2014;14.

47. Lili D, Ning L, Feili W, et al. Transmitted Antiretroviral Drug Resistance in the Men Who Have Sex with Men HIV Patient Cohort, Beijing, China, 2008-2011. *Viral Immunology.* Oct 2014;27(8):392-397.

48. Min C, Yanling M, Yingzhen S, et al. HIV-1 Genetic Characteristics and Transmitted Drug Resistance among Men Who Have Sex with Men in Kunming, China. *Plos One.* Jan 2014;9(1).

49. Bing S, Wen-Jing L, Ting L, et al. Subtype B Was the Dominant Strain Among HIV Type 1 Infections Except for the Population of Men Who Have Sex with Men in Harbin City, China. *Aids Research and Human Retroviruses.* Sep 2013;29(9):1260-1264.

50. Li L, Han N, Lu JF, et al. Genetic Characterization and Transmitted Drug Resistance of the HIV Type 1 Epidemic in Men Who Have Sex with Men in Beijing, China. *Aids Research and Human Retroviruses.* Mar 2013;29(3):633-637.

51. Hightow-Weidman LB, Lin H, He N, et al. Behavioral and molecular tracing of risky sexual contacts in a sample of Chinese HIV-infected men who have sex with men. *Journal of Medical Internet Research.* Feb 15 2013;177(4):343-350.

52. Lin L, Guoqing S, Tianyi L, et al. Multiple Introductions of HIV into Men Who Have Sex with Men Were Found in Zhengzhou City, China. *Aids Research and Human Retroviruses.* Sep 2012;28(9):947-951.

53. Zhao B, Han XX, Dai D, et al. New Trends of Primary Drug Resistance Among HIV Type 1-Infected Men Who Have Sex with Men in Liaoning Province, China. *Aids Research and Human Retroviruses.* Oct 2011;27(10):1047-1053.

54. Li L, Lu XL, Li HP, et al. High genetic diversity of HIV-1 was found in men who have sex with men in Shijiazhuang, China. *Infection Genetics and Evolution.* Aug 2011;11(6):1487-1492.

55. Guo HX, Wei JF, Yang HT, et al. Rapidly Increasing Prevalence of HIV and Syphilis and HIV-1 Subtype Characterization Among Men Who Have Sex With Men in Jiangsu, China. *Sexually Transmitted Diseases.* Feb 2009;36(2):120-125.

56. Wang WH, Jiang SL, Li SW, et al. Identification of Subtype B, Multiple Circulating Recombinant Forms and Unique Recombinants of HIV Type 1 in an MSM Cohort in China. *Aids Research and Human Retroviruses.* Oct 2008;24(10):1245-1254.

57. Zhang XY, Li SW, Li XX, et al. Characterization of HIV-1 subtypes and viral antiretroviral drug resistance in men who have sex with men in Beijing, China. *AIDS.* Dec 2007;21:S59-S65.

58. Maohe Y. *Analysis of behavior and HIV-1 subtypes, resistance among HIV-1 infected Men Who Have Sex in Tianjin* [D], Tianjin Medical University; 2011.

59. Chaojun Y, Li Y, Manhong J, et al. Analysis of HIV-1 subtype among men who have sex with men. *Modern Preventive Medicine.* 2011(04):686-688.

60. Xiaohui W, Yi B, Taiping H, et al. Epidemic strains of HIV-1 in MSM in Shenzhen :an cluster analysis. *Chinese Journal of Nosocomiology.* 2011;21(14):2892-2894.

61. Guanghua L. *The influence factors of HIV infection in a cohort of men who have sex with men in nanning city* [D], Guangxi Medical University; 2011.

62. Shu Z, Guozhang X, Fengjiao H, et al. Study on immunological status and viral subtype distribution of HIV-1 carriers in MSM, Ningbo. *China Preventive Medicine.* 2010(04):354-357.

63. Lihua L, Xianling P, Weihong L, et al. Study on the subtypes and sequence of HIV-1 strains in MSM. *Hebei Medical Journal.* 2010;32(3):287-288.

64. Zhiying L, Haiying L, Tong Z, et al. Molecular Epidemiologic Study on Humma Immunodeficiency Virus Type 1 Infection in Partial Men Who Have Sex with Men (MSM) in Beijing. *Journal of Capital Medical University.* 2009;30(5):616-620.

65. Xiujuan L, Lihua L, Hong Z, et al. Study on HIV-1 subtypes in men who have sex with men in Shijiazhuang. *Modern Preventive Medicine.* 2009;36(4):774-776,780.

66. Zhigang H, Huifang X, Yan L, et al. Molecular epidemiology of HIV-1 infection among MSM in Guangzhou. *Chinese Journal of Epidemiology.* 2009(04):381-384.

67. Guanglu Z, Tiejian F, Jin Z, et al. Study on molecular epidemiology of human immunodeficiency virus type 1 infection in men who have sex with men (MSM)in Shenzhen. *CHINESE Journal of AIDS & STD.* 2008;14(2):137-141.

68. Xiaoxu H. Analysis on primary drug resistance of HIV-1 infected man having sex with man. *Chinese Journal of Public Health.* 2007;23(12).
