# Supplementary material for: The prevalence, temporal trends, and geographical distribution of HIV-1 subtypes among men who have sex with men in China: A systematic review and meta-analysis
Source: Epidemiol Infect. 2019 Feb 19;147:e83. doi: 10.1017/S0950268818003400 (PMC6518548; doi:10.1017/S0950268818003400)
Supplement: Supplementary file 1 [file S0950268818003400sup001.zip › S0950268818003400sup001/TableS2.docx]

Table S2. Temporal trends of different HIV-1 subtypes among MSM in China

|  | <=2007 | | |  | 2008-2010 | | |  | 2011-2013 | | |  | >=2014 | | | P# |
| --- | --- | --- | --- | --- | --- | --- | --- | --- | --- | --- | --- | --- | --- | --- | --- | --- |
|  | n | proportion(%)  (95%CI) | p*,I^2^ |  | n | proportion(%)  (95%CI) | p*,I^2^ |  | n | proportion(%)  (95%CI) | p*,I^2^ |  | n | proportion(%)  (95%CI) | p*,I^2^ |  |
| CRF01_AE | 9 | 45.16  (31.11,57.48) | <0.01, 73.9% |  | 18 | 57.62  (25.54,62.62) | <0.01, 74.0% |  | 25 | 58.82  (50.01,62.58) | <0.01, 77.9% |  | 8 | 58.46  (72.26,69.24) | <0.01, 89.7% | 0.34 |
| CRF07_BC | 7 | 9.09  (40.03,15.54) | 0.12, 40.0% |  | 15 | 15.29  (96.66,21.82) | <0.01, 84.6% |  | 24 | 25.55  (17.78,29.49) | <0.01, 83.5% |  | 8 | 21.13  (22.23,31.64) | <0.01, 90.9% | < 0.01 |
| B | 9 | 41.91  (85.58,55.82) | <0.01, 79.7% |  | 15 | 25.94  (91.18,33.28) | <0.01, 89.8% |  | 24 | 9.32  (73.34,11.50) | <0.01, 72.2% |  | 6 | 9.20  (24.43,19.28) | <0.01, 90.9% | < 0.01 |
| CRF08_BC | 2 | 7.57  (0.00,25.84) | 0.09, 65.4% |  | 5 | 4.63  (12.25,9.64) | <0.01, 79.2% |  | 4 | 2.99  (00.06,8.74) | <0.01, 90.6% |  | 1 | 1.53  (00.02,4.55) |  | < 0.01 |
| CRF01_B |  |  |  |  |  |  |  |  | 11 | 3.97  (19.92,6.61) | <0.01, 87.9% |  | 1 | 4.76  (06.61,11.72) |  | 0.93 |
| C |  |  |  |  | 5 | 1.56  (08.81,2.51) | 0.54, 0.0% |  | 2 | 1.14  (00.04,3.14) | 0.40, 0.0% |  | 2 | 2.74  (0.00,10.41) | 0.05, 73.0% | < 0.01 |
| URFs | 1 | 20.69  (75.59,37.60) |  |  | 2 | 1.03  (02.26,2.16) | 0.50, 0.0% |  | 10 | 4.70  (30.07,6.61) | 0.09, 40.5% |  | 6 | 3.94  (13.39,7.49) | 0.01, 64.6% | < 0.01 |
| CRF55_01B |  |  |  |  |  |  |  |  | 8 | 1.81  (07.73,3.27) | <0.01, 76.9% |  | 1 | 4.76  (06.61,11.72) |  | 0.14 |

N: Number of estimates

* P value was calculated by examining Cochran’s Q statistics (P<0.1 was considered statistically significant).

# P value stand for the differences between groups (P<0.05 was considered statically significant).
