# Supplementary material for: The prevalence, temporal trends, and geographical distribution of HIV-1 subtypes among men who have sex with men in China: A systematic review and meta-analysis
Source: Epidemiol Infect. 2019 Feb 19;147:e83. doi: 10.1017/S0950268818003400 (PMC6518548; doi:10.1017/S0950268818003400)
Supplement: Supplementary file 1 [file S0950268818003400sup001.zip › S0950268818003400sup001/TableS3.docx]

Table S3. Prevalence of HIV-1 subtypes in different area among MSM in China.

|  | East | | |  | North | | |  | Northeast | | |  | Southwest | | | P# |
| --- | --- | --- | --- | --- | --- | --- | --- | --- | --- | --- | --- | --- | --- | --- | --- | --- |
|  | n | proportion(%)  (95%CI) | P*,I^2^ |  | n | proportion(%)  (95%CI) | P*,I^2^ |  | n | proportion(%)  (95%CI) | P*,I^2^ |  | n | proportion(%)  (95%CI) | P*,I^2^ |  |
| CRF01_AE | 28 | 56.64  (51.15-62.02) | <0.01, 92.30% |  | 29 | 50.64  (47.08-54.21) | <0.01, 68.00% |  | 8 | 78.78  (72.45-84.52) | 0.01, 60.30% |  | 3 | 74.87  (60.87-86.8) | 0.07, 62.30% | < 0.01 |
| CRF07_BC | 27 | 27.53  (23.7-31.53) | <0.01, 86.50% |  | 23 | 16.30  (11.72-21.43) | <0.010, 88.80% |  | 8 | 5.73  (3.31-8.65) | 0.20, 29.10% |  | 3 | 15.02  (4.29-29.81) | 0.030, 72.40% | < 0.01 |
| B | 26 | 9.95  (7.28-12.96) | <0.01, 87.60% |  | 27 | 28.15  (21.98-34.75) | <0.010, 92.70% |  | 7 | 10.83  (7.11-15.15) | 0.10, 43.60% |  | 1 | 3.05  (1.16-8.01) |  | < 0.01 |
| CRF08_BC | 8 | 1.16  (0.09-2.98) | <0.01, 82.80% |  | 3 | 4.33  (0.00-18.01) | <0.01, 91.00% |  | 1 | 19.35  (7.05-35.39) |  |  | 2 | 4.64  (0.00-16.97) | 0.02, 83.00% | < 0.01 |
| CRF01_B | 11 | 4.83  (2.54-7.75) | <0.01, 91.50% |  | 6 | 3.95  (0.71-9.11) | <0.010, 88.10% |  |  |  |  |  |  |  |  | 0.85 |
| C | 6 | 1.36  (0.19-3.29) | <0.01, 82.50% |  | 4 | 0.98  (0.17-2.22) | 0.810, 0.00% |  |  |  |  |  |  |  |  | 0.87 |
| URFs | 6 | 2.54  (1.54-3.73) | 0.38, 5.80% |  | 10 | 4.53  (2.14-7.62) | <0.01, 82.30% |  | 3 | 4.85  (2.10-8.47) | 0.56, 0.00% |  | 1 | 5.34  (2.04-9.96) |  | 0.36 |
| CRF55_01B | 8 | 4.27  (1.48-8.29) | <0.01, 95.00% |  | 4 | 0.83  (0.03-2.29) | 0.110, 49.90% |  |  |  |  |  |  |  |  | 0.04 |

* P value was calculated by examining Cochran’s Q statistics (P<0.1 was considered statistically significant).

# P value stand for the differences between groups (P<0.05 was considered statically significant).
