# Supplementary material for: The prevalence, temporal trends, and geographical distribution of HIV-1 subtypes among men who have sex with men in China: A systematic review and meta-analysis
Source: Epidemiol Infect. 2019 Feb 19;147:e83. doi: 10.1017/S0950268818003400 (PMC6518548; doi:10.1017/S0950268818003400)
Supplement: Supplementary file 1 [file S0950268818003400sup001.zip › S0950268818003400sup001/TableS4.docx]

Table S4. Subgroup analysis by different amplification HIV-1 region.

|  | A | | |  | B | | |  | C | | | P |
| --- | --- | --- | --- | --- | --- | --- | --- | --- | --- | --- | --- | --- |
|  | n | proportion(%)(95%CI) | Q, I2 |  | n | proportion(%)(95%CI) | Q, I2 |  | n | proportion(%)(95%CI) | Q, I2 |  |
| CRF01_AE | 37 | 59.99(54.80,65.07) | 507.03, 92.9% |  | 20 | 58.01(52.33,63.59) | 112.09, 83.0% |  | 10 | 46.48(39.27,53.76) | 34.6, 74.0% | 0.0097 |
| CRF07_BC | 34 | 18.32(14.22,22.80) | 453.28, 92.7% |  | 19 | 21.10(15.18,27.65) | 184.35, 90.2% |  | 8 | 20.73(14.02,28.33) | 30.28, 76.9% | 0.7197 |
| B | 32 | 16.73(12.40,21.53) | 559.27, 94.5% |  | 19 | 13.21(9.83,16.96) | 82.16, 78.1% |  | 10 | 27.16(13.67,43.13) | 192.1, 95.3% | 0.1143 |
| CRF08_BC | 8 | 2.50(0.33,6.00) | 54.57, 87.2% |  | 5 | 3.48(0.74,7.77) | 28.34, 85.9% |  | 1 | 1.53(0.02,4.55) | 0, -- | 0.579 |
| CRF01_B | 9 | 4.79(2.06,8.44) | 139.61, 93.6% |  | 5 | 3.07(0.54,7.21) | 31.28, 87.2% |  | 3 | 7.19(2.18,14.54) | 3.09, 67.7% | 0.461 |
| C | 5 | 1.05(0.00,3.40) | 17.51, 77.2% |  | 4 | 1.87(0.99,2.97) | 1.33, 0.0% |  | 1 | 0.61(0.00,2.60) | 0, -- | 0.4537 |
| URFs | 9 | 2.77(1.44,4.46) | 22.12, 63.8% |  | 7 | 3.73(2.21,5.57) | 7.09, 15.4% |  | 4 | 8.61(4.19,14.27) | 8.04, 62.7% | 0.0342 |
| CRF55_01B | 7 | 2.53(0.35,6.29) | 145.47, 95.9% |  | 3 | 3.68(0.00,12.37) | 27.63, 92.8% |  | 2 | 4.54(0.00,16.36) | 8.68, 88.5% | 0.8472 |

A=HIV-1 genotyped by only one region. (env or gag or pol)

B= HIV-1 genotyped by two regions. (env&gag or env&pol or gag&pol)

C=HIV-1 genotyped by three regions (env&geg&pol)
