# Supplementary figures and images for: The prevalence, temporal trends, and geographical distribution of HIV-1 subtypes among men who have sex with men in China: A systematic review and meta-analysis
Source: Epidemiol Infect. 2019 Feb 19;147:e83. doi: 10.1017/S0950268818003400 (PMC6518548; doi:10.1017/S0950268818003400)

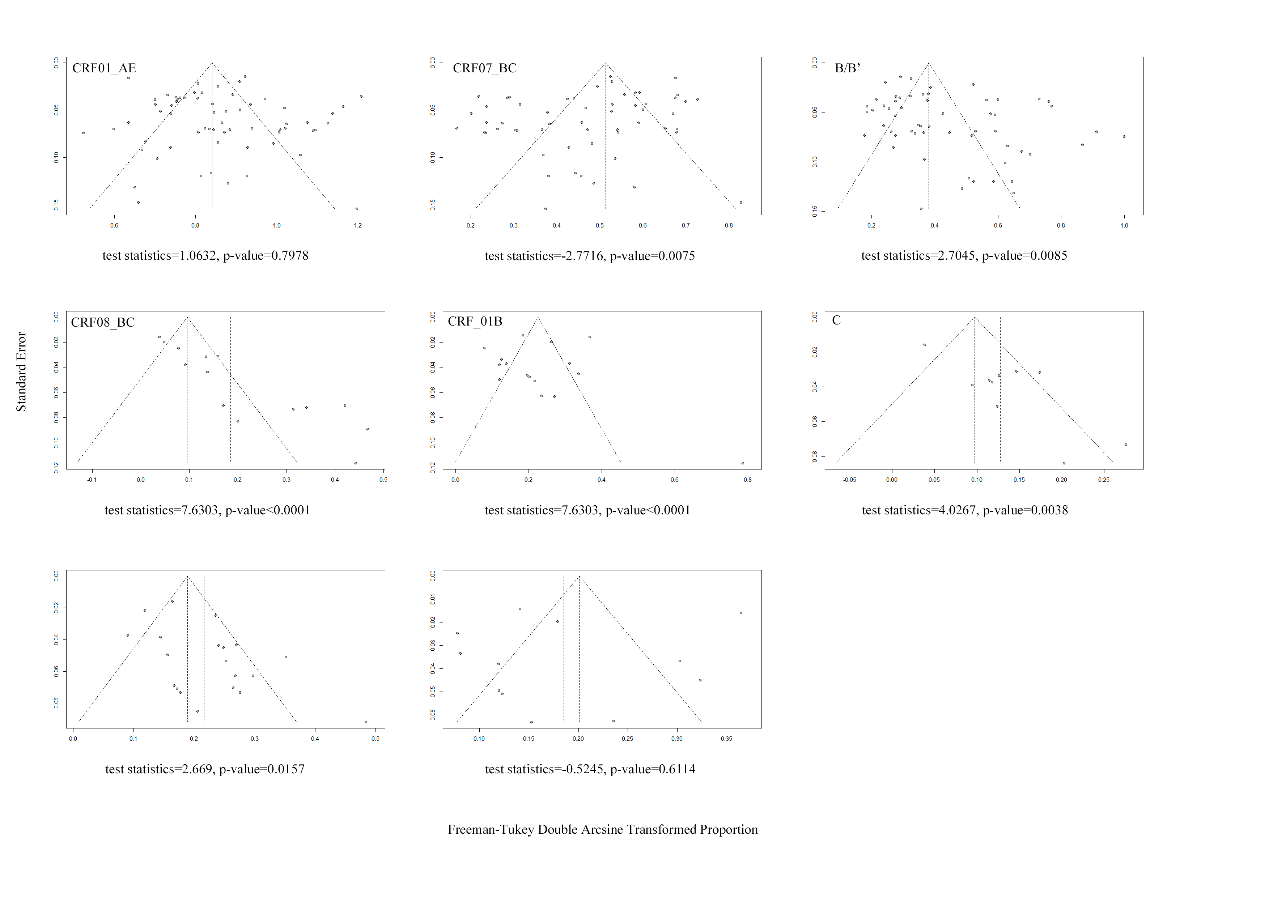


Figure S1: Funnel plot and Egger’s test of different HIV-1 subtypes among MSM in China.

Supplement: Supplementary file 1 [file S0950268818003400sup001.zip › S0950268818003400sup001/FigureS1.docx]

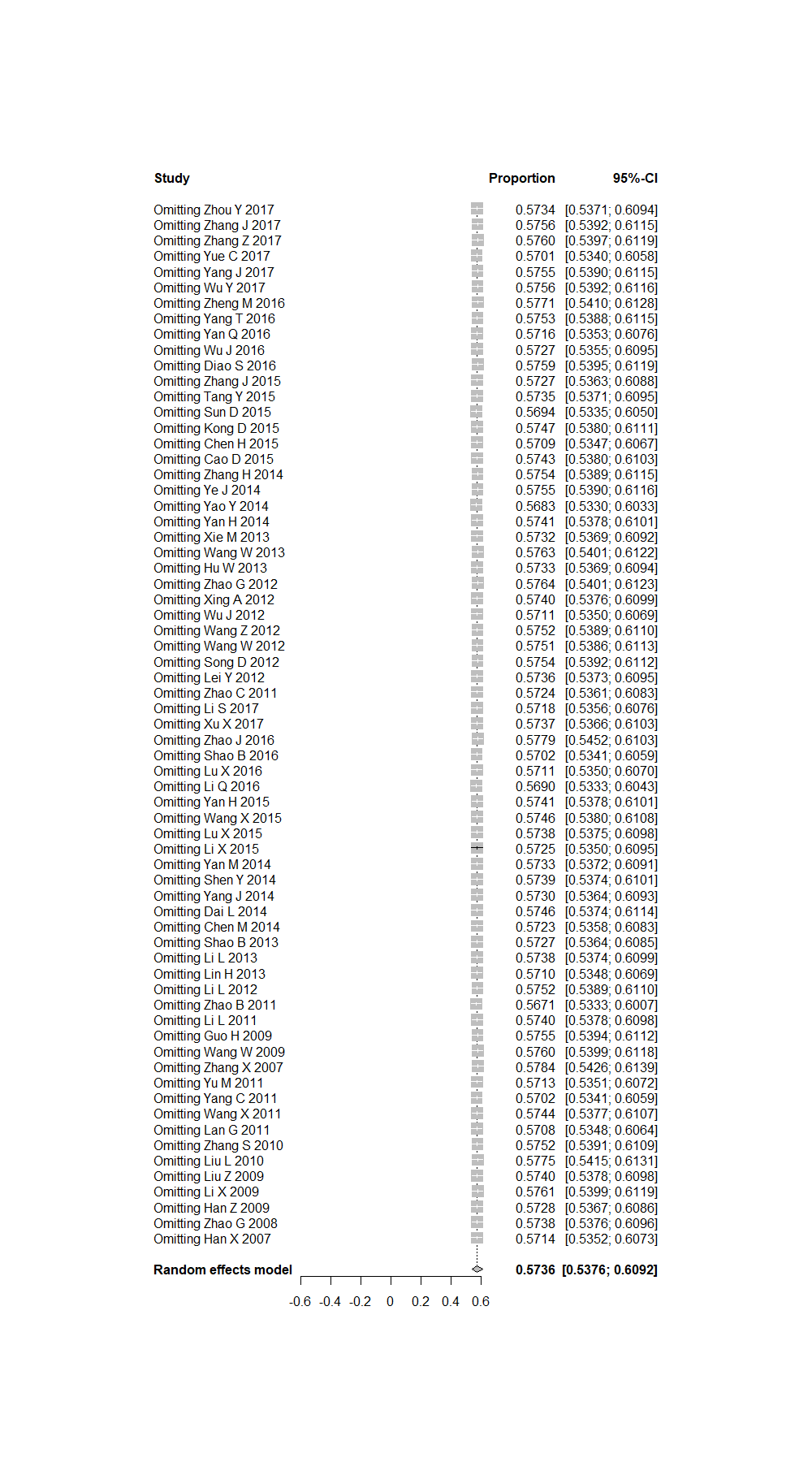


Figure S2: The forest plot of sensitivity analysis of the proportion of CRF01_AE.

Supplement: Supplementary file 1 [file S0950268818003400sup001.zip › S0950268818003400sup001/FigureS2.docx]
